# Nationwide epidemiological survey of autoimmune pancreatitis in Japan in 2021

**DOI:** 10.1007/s00535-026-02438-w

**Published:** 2026-05-19

**Authors:** Atsushi Masamune, Takanori Sano, Tetsuya Takikawa, Ryotaro Matsumoto, Shin Hamada, Toshiaki Abe, Yuichi Tanaka, Yoshifumi Takeyama, Kazuhiro Kikuta, Japan Pancreatitis Study Group, Takahiro Abe, Makoto Abue, Takumi Akiyama, Toshihiko Arizumi, Shohei Asada, Shunjiro Azuma, Hiroyoshi Doi, Takaaki Eguchi, Nao Fujimori, Akashi Fujita, Akihisa Fukuda, Yoshihisa Fukuda, Hiroyuki Funatyama, Toshifumi Gushima, Sumi Hajime, Yasushi Hamaya, Kiyotaka Hashizume, Ayako Hata, Nobuhiro Hattori, Kazuki Hayashi, Kazunao Hayashi, Koki Hayashi, Nobuhiko Hayashi, Itagaki Hideki, Kawakami Hiroshi, Morihisa Hirota, Endo Hiroyuki, Kazunari Ibusuki, Akihito Ida, Takao Iemoto, Takeshi Iida, Sadaharu Iio, Tsukasa Ikeura, Tsunao Imamura, Tomoko Inagaki, Osamu Inatomi, Kentaro Inoue, Kazunori Ishige, Akio Ishii, Shuichi Ito, Takashi Ito, Tetsuhide Ito, Eisuke Iwasaki, Takuji Iwashita, Keisuke Iwata, Junji Kawaguchi, Naruomi Jinno, Takuo Kado, Seiji Kaino, Masatoshi Kajiwara, Toyoma Kaku, Keiko Kaneko, Atsushi Kanno, Kobashigawa Kasen, Satoshi Kasugai, Shin Kato, Shinya Kawaguchi, Yuki Kawaji, Toru Kawamoto, Nagata Kazuyoshi, Osamu Kido, Yui Kishimoto, Hideki Kitada, Takanori Kitaguchi, Rena Kitano, Masanori Kobayashi, Ikuhiro Kobori, Yuzo Kodama, Kazuhiko Koike, Mitsuhito Koizumi, Shunsuke Komoto, Naoki Konno, Yudai Koya, Kanemitsu Kozue, Atsushi Kubo, Yoshimasa Kubota, Kiyoshi Kume, Hitoshi Kurata, Michio Kuroki, Masaki Kuwatani, Isamu Makino, Toru Maruo, Akinori Maruta, Saburo Matsubara, Kazuyuki Matsumoto, Susumu Matsuo, Ichiro Miyajima, Masaki Miyazawa, Suguru Mizuno, Toshihiro Morita, Shuntaro Mukai, Yoshiyuki Murawaki, Ryoji Nagamura, Kazunari Nakahara, Yousuke Nakai, Akira Nakamura, Kazunori Nakaoka, Hiroshi Nakase, Atsushi Nakazawa, Hyoda Naoko, Yusuke Niina, Tsutomu Nishida, Takayoshi Nishino, Shotaro Noge, Hideharu Ogiyama, Takaya Oguchi, Akihiko Ohata, Tomoyuki Ohno, Masahiro Ohtani, Hiroshi Ohyama, Hiromitsu Oka, Shiro Oka, Toshimasa Okada, Yasuyuki Okada, Hiromasa Okamoto, Kazuichi Okazaki, Fumihiro Okumura, Kosuke Okuwaki, Takao Oyama, Nishio Ryo, Kei Saito, Michihiro Saito, Nozomu Saito, Toshitaka Sakai, Yoshitaka Sakai, Naoki Sasahira, Yutaka Sasaki, Yuichi Sasakura, Masahiro Sato, Satoshi Sato, Yoichiro Sato, Akihiko Satoh, Yugo Sawai, Masanari Sekine, Minoru Shigekawa, Masahiro Shiihara, Yutaka Shimamatsu, Masaaki Shimatani, Takehiro Shimizu, Nobuhiko Shinohara, Hideyuki Shiomi, Yoshio Sogame, Takahiro Suda, Shigeyuki Suenaga, Kaoru Suzuki, Noriaki Suzuki, Rei Suzuki, Yuhei Suzuki, Akinari Tabaru, Kosuke Takagaki, Tadayuki Takagi, Kenichi Takahashi, Kenji Takahashi, Seiichi Takahashi, Dohmen Takahiro, Shinichi Takano, Yuichi Takano, Yusuke Takasaki, Mamoru Takenaka, Eiji Tamoto, Yu Tanaka, Shiroh Tanoue, Shuji Terai, Masao Toki, Yuichi Torisu, Izumi Toshinobu, Takayuki Tsujikawa, Masahiro Tsujimae, Hidehiko Tuda, Kazushige Uchida, Shinya Uemura, Masayuki Ueno, Yoshiyuki Ueno, Jun Unno, Shigeki Wakiyama, Kenta Yamada, Reiko Yamada, Akira Yamamiya, Satoshi Yamamoto, Kohei Yamanouchi, Kentaro Yamao, Hiroaki Yasuda, Ichiro Yasuda, Tatsuji Yogi, Murayama Yoko, Tomoyuki Yokota, Hideo Yoshida, Naoki Yoshida, Hayato Yoshimura, Reiko Yoshioka

**Affiliations:** 1 Division of Gastroenterology, Tohoku University Graduate School of Medicine, 1-1 Seiryo-machi, Aoba-ku, Sendai, 980-8574 Japan; 2 Osaka Gyoumeikan Hospital, Osaka, Japan

**Keywords:** Autoimmune pancreatitis, EUS-FNA, EUS-TA, Glucocorticoid, IgG4-related disease

## Abstract

**Background:**

To update the clinic-epidemiological features of autoimmune pancreatitis (AIP) in Japan, we conducted the fifth nationwide survey.

**Methods:**

We performed a two-stage survey of patients with AIP who were treated at selected hospitals in 2021. The first-stage survey estimated the total number of patients with AIP, and the second-stage survey analyzed detailed clinical characteristics.

**Results:**

The estimated number of patients with AIP in 2021 was 16,750 (prevalence, 13.3 per 100,000 persons), representing a 25% increase from 2016. The number of newly diagnosed cases was 4210 (annual incidence, 3.4 per 100,000 persons). Clinical data were available for 2833 patients. The male-to-female ratio was 3.32:1, and the mean age in 2021 and the mean age at AIP diagnosis were 71.2 and 66.3 years, respectively. At diagnosis, 58.6% of patients were symptomatic, approximately half of whom presented with jaundice. Other organ involvement was observed in 67.8% of patients, most commonly sclerosing cholangitis. Histopathological examination was performed in 84.1% of cases, predominantly using endoscopic ultrasonography-guided tissue acquisition. Initial glucocorticoid therapy was administered to 84.2% of patients, and 93.8% received maintenance therapy. Kaplan–Meier analysis demonstrated a cumulative relapse incidence of 12.5%, 20.7%, 36.0%, 44.0%, 53.3%, and 68.9% at 3, 5, 10, 15, 20, and 25 years, respectively. Long-term overall survival was favorable; however, malignancies accounted for 40% of deaths.

**Conclusions:**

This nationwide survey updates the epidemiological and clinical characteristics of AIP in Japan. Patient numbers continue to rise, and the high cumulative risk of relapse underscores the need for second-line therapies beyond glucocorticoids.

**Supplementary Information:**

The online version contains supplementary material available at https://doi.org/10.1007/s00535-026-02438-w.

## Introduction

Autoimmune pancreatitis (AIP) is a relatively rare form of pancreatitis with a presumed autoimmune pathogenesis [1–4]. Since its first description by Yoshida et al. in 1995 [5], AIP has become established as a distinct disease entity. Several diagnostic criteria have been proposed, including the International Consensus Diagnostic Criteria (ICDC) [6] and the revised clinical diagnostic criteria proposed by the Japan Pancreas Society in 2018 (JPS2018 criteria) [7]. AIP is characterized by distinct clinical, serological, morphological, and histological features [1–4]. AIP comprises two distinct subtypes: type 1 and type 2. Histologically, lymphoplasmacytic sclerosing pancreatitis is characteristic of type 1 AIP, whereas idiopathic duct-centric pancreatitis with granulocytic epithelial lesions is characteristic of type 2 AIP [1–4]. Type 1 AIP is regarded as the pancreatic manifestation of systemic immunoglobulin G4-related disease (IgG4-RD) and is frequently associated with elevated serum IgG4 levels [8] and extrapancreatic involvement, including IgG4-related sclerosing cholangitis, sialadenitis/dacryoadenitis, and retroperitoneal fibrosis [1–4]. Diffuse pancreatic enlargement and irregular narrowing of the main pancreatic duct (MPD) are characteristic imaging features of AIP.

In Japan, nationwide epidemiological surveys of pancreatitis, including AIP, have been conducted every 4–5 years [9–12]. The first nationwide survey for AIP analyzed patients in 2002 [9], followed by subsequent surveys in 2007 [10], 2011 [11], and 2016 [12]. The estimated number of patients with AIP increased from 1700 in 2002 to 2790 in 2007, 5745 in 2011, and 13,436 in 2016 [9–12]. In addition to estimating prevalence and incidence, these surveys have clarified the clinical characteristics of AIP. Three decades after the initial description, we conducted a fifth nationwide survey to provide updated epidemiological and clinicopathological data and to assess long-term outcomes of patients with AIP in Japan.

## Methods

We conducted a two-stage survey. The first-stage survey aimed to estimate the prevalence and incidence of AIP in Japan, whereas the second-stage survey aimed to characterize its clinical features and long-term outcomes.

### Diagnosis of AIP

AIP was diagnosed according to the JPS2018 criteria [7], which comprise six major components: pancreatic enlargement, irregular narrowing of MPD, elevated serum IgG4 levels, histopathological findings, other organ involvement (OOI), and responsiveness to glucocorticoid (GC) therapy. The JPS2018 criteria define IgG4-related sclerosing cholangitis, sialadenitis/dacryoadenitis, retroperitoneal fibrosis, and IgG4-related kidney disease as OOI. The JPS2018 criteria are primarily intended for the diagnosis of type 1 AIP, whereas the ICDC are required for the diagnosis of type 2 AIP or AIP-not otherwise specified [6, 7].

### First-stage survey

The target population comprised patients with AIP treated at selected hospitals in 2021. Hospitals were identified using the *Hospital Yearbook 2021* (R&D Co., Ltd., Nagoya, Japan), and a list of critical care and emergency centers was obtained from the website of the Japanese Association for Acute Medicine (https://www.jaam.jp/about/shisetsu/qq-center.html). Departments of internal medicine, gastroenterology, surgery, and digestive surgery were selected and subjected to stratified random sampling.

Sampling rates were 5%, 10%, 20%, 40%, 80%, and 100% for hospitals with ≤ 99, 100–199, 200–299, 300–399, 400–499, and ≥ 500 beds, respectively. All affiliated university hospitals were included. Because the survey also included patients with acute pancreatitis [13], critical care and emergency centers were treated as a special stratum, and all were included (Supplementary Table 1).

In July 2022, a questionnaire inquiring about the number of patients with AIP who were treated at each hospital in 2021 was mailed to 2398 randomly selected departments. Responses were subsequently collected online. The total number of patients with AIP was estimated under the assumption that response probability was independent of patient volume. The formulae used to calculate the estimated number of patients and corresponding 95% confidence intervals have been described previously [12, 13]. Prevalence and annual incidence were calculated using the Japanese population in 2021 (*n* = 125,502,000).

### Second-stage survey

In July 2023, a second-stage questionnaire was sent to departments that had reported treating patients with AIP in the first-stage survey. Responses were collected online through March 2024. Because some items were incomplete, the number of patients included in each analysis varied.

### Statistical analysis

Continuous variables are presented as mean ± standard deviation (SD) or median (interquartile range [IQR]), as appropriate. Continuous variables were compared using the unpaired Student’s *t*-test. Categorical variables were compared using the Chi-square test or Fisher’s exact test, as appropriate. Sex differences in the prevalence of OOI were assessed using the Chi-square test. Survival curves were estimated using the Kaplan–Meier method and compared using the log-rank test. All tests were two-sided, and *P* < 0.05 was considered statistically significant. Statistical analyses were performed using SPSS Statistics version 20.0 (SPSS Inc., Chicago, IL, USA).

### Ethics

This study was approved by the Ethics Committee of the Tohoku University Graduate School of Medicine (approval numbers: 2022-1-050 and 2023-1-732-1). The requirement for informed consent was waived because of the retrospective nature of the study. Study information was made available on the institutional website, and patients were given the opportunity to opt out.

## Results

### First-stage survey

**Fig. 1:**
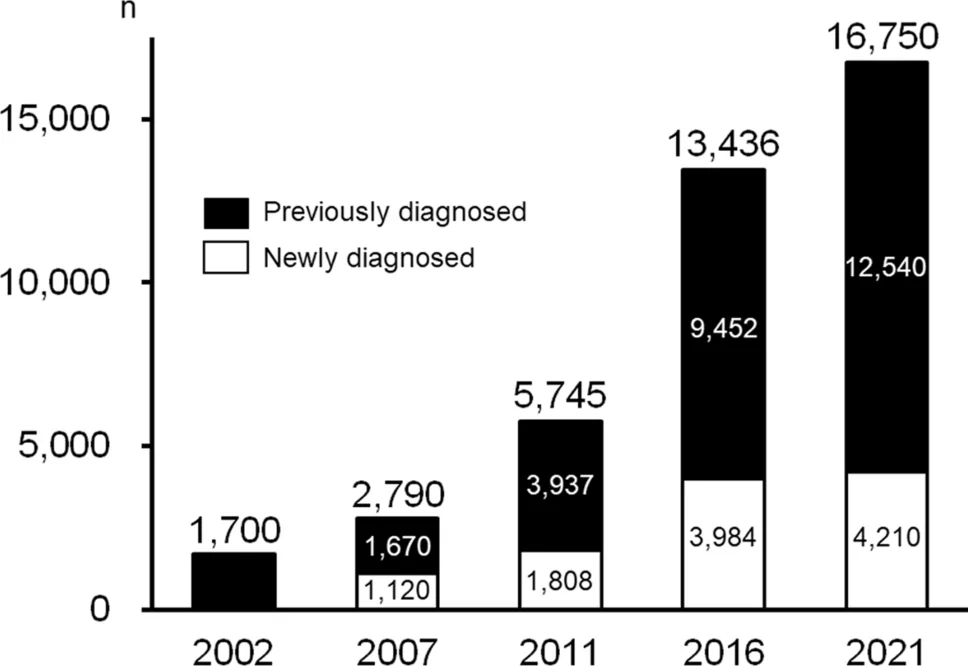
Changes in the estimated number of patients with AIP in Japan. Estimated numbers of patients with AIP in Japan, including both previously diagnosed and newly diagnosed cases, are shown for the previous surveys (2002, 2007, 2011, and 2016) and the current survey (2021)

The results of the first-stage survey are summarized in Supplementary Table 1. Of the 2398 departments surveyed, 877 responded (response rate, 36.6%), reporting 5971 patients with AIP. The estimated total number of patients with AIP in Japan was 16,750 (95% confidence interval, 15,500–17,990), corresponding to a prevalence of 13.3 per 100,000 persons. The estimated number of newly diagnosed patients was 4210 (95% confidence interval, 3650–4760), with an annual incidence of 3.4 per 100,000 persons. These estimates have increased steadily since 2002 (Fig. 1). Compared with the 2016 survey [12], the estimated total number of patients and newly diagnosed cases increased by 1.25-fold and 1.06-fold, respectively.

### Second-stage survey

**Fig. 2:**
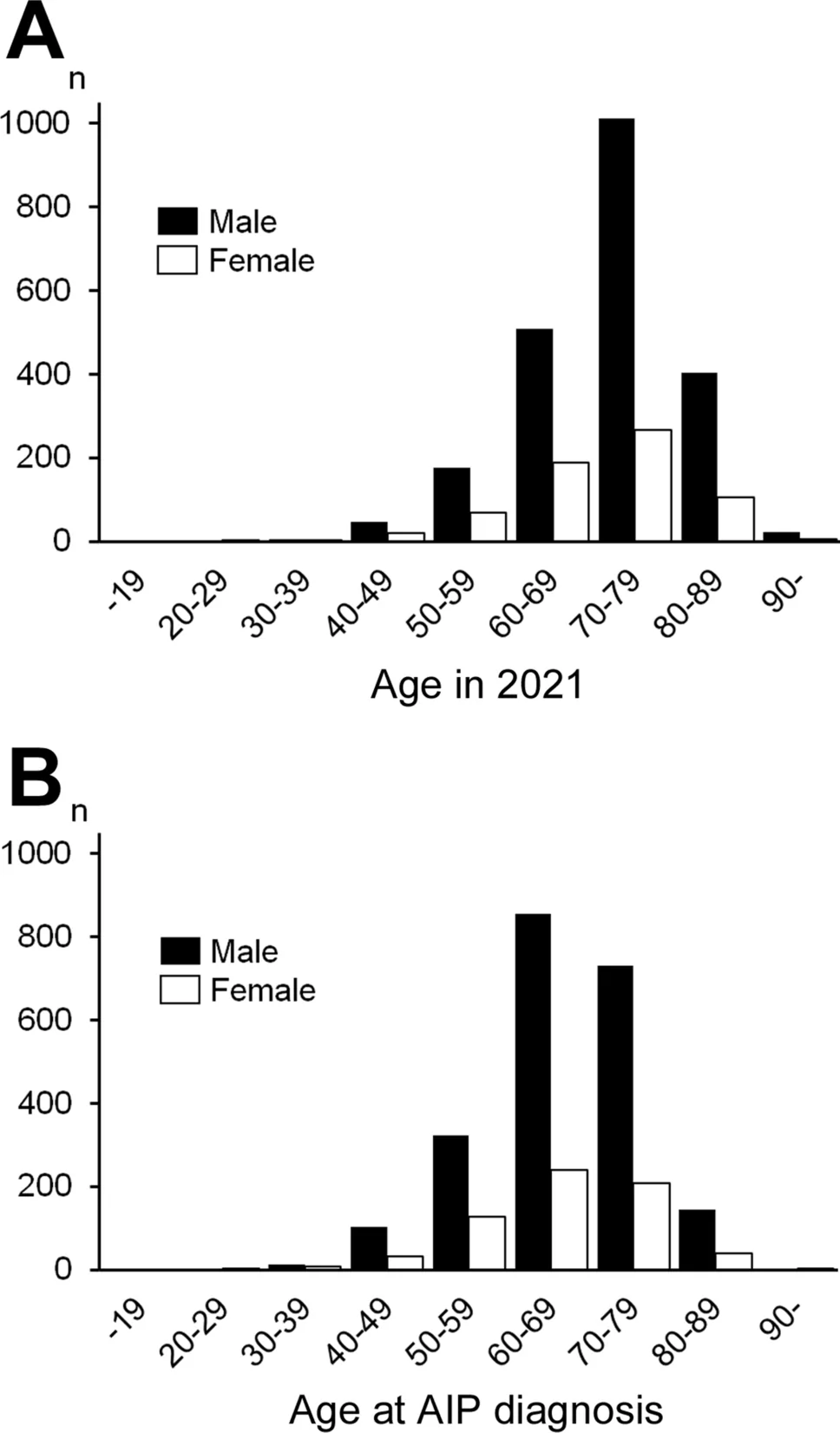
Age distribution of patients with AIP. Age in 2021 (**A**) and age at diagnosis (**B**) of patients with AIP, stratified by sex

Detailed clinical data were obtained for 2833 (2177 males and 656 females) patients with definite (*n* = 2404) or probable (*n* = 429) AIP. The male-to-female ratio was 3.32:1. The mean (SD) age in 2021 was 71.2 (9.6) years (71.6 [9.5] years in males and 70.1 [10.0] years in females), and the mean (SD) age at AIP diagnosis was 66.3 (9.8) years (66.5 [9.7] in males and 65.6 [10.2] years in females) (Fig. 2). The peak age groups were 70–79 years for age in 2021 and 60–69 years for age at AIP diagnosis in both sexes.

#### Clinical presentation

Clinical presentation data were available for 2810 patients, of whom 1648 (58.6%) were symptomatic at diagnosis. Jaundice was the most common presenting symptom, accounting for approximately half of symptomatic cases, followed by abdominal pain (23.9%) (Supplementary Table 2). Symptoms related to extrapancreatic involvement were present in 8.1% of patients. Acute pancreatitis at presentation was uncommon (2.1%). Among asymptomatic patients, 804 (28.6%) were identified during follow-up or workup for other diseases, and 304 (10.8%) through medical check-ups.

#### Serology

Serum IgG4 levels at AIP diagnosis were available for 2792 patients, with a median (IQR) of 528.6 (203.3–671.5) mg/dL. Elevated IgG4 levels (≥ 135 mg/dL) were observed in 2535 of 2792 patients (90.8%) (Supplementary Fig. 1). Elevated IgG levels (> 1700 mg/dL) were observed in 1437 of 2621 patients (54.8%). Antinuclear antibodies and rheumatoid factor were positive in 442 of 1521 patients (29.1%) and 202 of 935 patients (21.6%), respectively.

#### Imaging findings

Pancreatic enlargement was observed in 99.5% of patients and MPD narrowing in 88.1% (Supplementary Table 3). Diffuse pancreatic enlargement and diffuse MPD narrowing were observed in 55.8% and 42.2% of patients, respectively. MPD narrowing was evaluated by endoscopic retrograde cholangiopancreatography alone in 10.9%, magnetic resonance cholangiopancreatography alone in 37.4%, and both modalities in 49.6%.

#### Histopathology

**Fig. 3:**
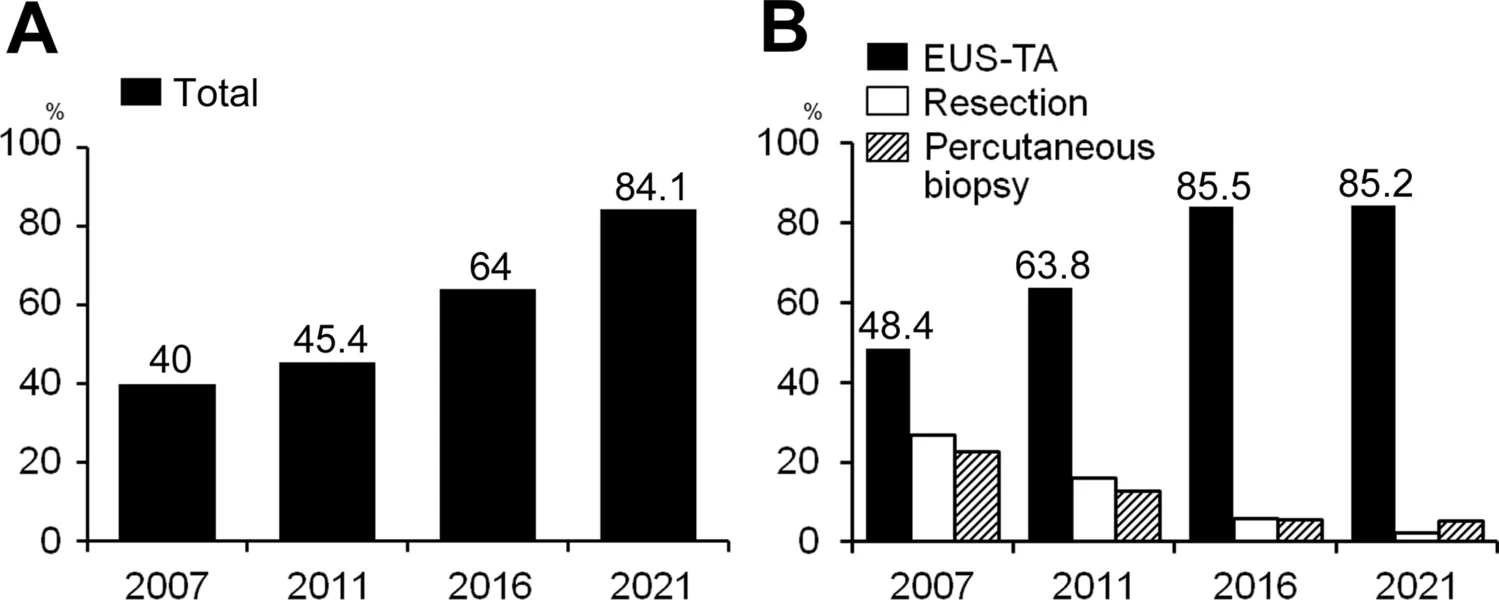
Changes in the proportion and procedures for pancreatic tissue sampling. The proportion of patients undergoing pancreatic tissue sampling (“Total”) is shown in panel A, and the procedures used for tissue sampling are shown in panel B for the previous and current surveys. The proportion of patients undergoing EUS-guided sampling increased markedly over time (48.4% in 2007, 63.8% in 2011, 85.5% in 2016, and 85.2% in 2021). Conversely, the proportions undergoing surgical resection or percutaneous biopsy decreased substantially (resection: 26.8% in 2007 to 2.3% in 2021; percutaneous biopsy: 22.6% to 4.0%). EUS-TA, endoscopic ultrasonography-guided tissue acquisition

Histopathological data were available for 2782 patients, and tissue sampling was performed in 2340 (84.1%). Among 2317 patients with documented procedures, endoscopic ultrasonography-guided tissue acquisition (EUS-TA) was used in 1973 (85.2%), followed by endoscopic retrograde cholangiopancreatography-guided sampling (5.4%) (Supplementary Table 4). The proportion of patients undergoing histological examination increased over time (40% in 2007, 45.4% in 2011, 64% in 2016, and 84.1% in 2021) (Fig. 3A). The use of EUS-guided sampling increased markedly (48.4–85.2%), whereas surgical resection and percutaneous biopsy declined substantially [9–12] (Fig. 3B). Among 2293 patients with evaluable histology, 533 (23.2%) fulfilled criterion IVa and 583 (25.4%) fulfilled criterion IVb of the JPS2018 criteria.

#### OOI

OOI as defined in the JPS2018 criteria was observed in 1911 of 2819 patients (67.8%) (Table 1). IgG4-related sclerosing cholangitis was the most common manifestation, followed by sialadenitis/dacryoadenitis and retroperitoneal fibrosis (Table 1). The distribution of OOI differed by sex; lacrimal and salivary gland involvement was more frequent in females, whereas sclerosing cholangitis, retroperitoneal fibrosis, and aortitis/periaortitis were more common in males (Supplementary Table 5).

**Table 1:** Distribution of OOI observed in patients with AIP

Type of OOI	*n* (%)^a^
IgG4-related sclerosing cholangitis	1363 (48.4)
Proximal	306 (10.9)^b^
Distal	1234 (43.8)^b^
Sialadenitis/dacryoadenitis	566 (20.1)
Sialadenitis	489 (17.3)^c^
Dacryoadenitis	205 (7.3)^c^
Retroperitoneal fibrosis	342 (12.1)
IgG4-related kidney disease	235 (8.3)
IgG4-related lung disease	162 (5.7)
Aortitis/periaortitis	133 (4.7)
IgG4-related cholecystitis	70 (2.5)
Pseudotumor	64 (2.3)
IgG4-related liver disease	25 (0.9)
Total cases with any OOI^d^	1968 (69.8)
Total cases with any OOI defined in the JPS2018	1911 (67.8)
Number of OOI per patient, mean (SD)^e^	0.89 (0.77)
4	11 (0.4)
3	71 (2.5)
2	420 (14.9)
1	1,409 (50.0)

*OOI* other organ involvement, *SD* standard deviation

^a^Among 2819 cases

^b^Overlap observed in 177 cases

^c^Overlap observed in 128 cases

^d^OOI listed in this table

^e^OOI defined in the JPS2018 criteria

#### Treatment

Initial GC therapy was administered to 2383 of 2831 patients (84.2%). The starting dose of prednisolone (PSL) was available for 2253 patients, with 30 mg/day being the most common dose, used in 1214 patients (53.9%) (Supplementary Fig. 2A). Among 1892 patients with available body weight data, 714 patients (37.7%) received the guideline-recommended dose (approximately 0.6 mg/kg/day) (Supplementary Fig. 2B). Almost all patients (2376/2380; 99.8%) responded to initial GC therapy. Maintenance therapy was administered to 2226 of 2374 patients (93.8%). Immunomodulatory agents were used in 3.4% of patients, most commonly azathioprine (Supplementary Table 6).

Diabetes mellitus was present in 1250 of 2790 patients (44.8%) before initial GC therapy and increased significantly to 1428 (51.2%) after therapy (*P* < 0.001). Similarly, pancreatic exocrine insufficiency was observed in 409 of 2813 patients (14.5%) before treatment and increased to 476 (16.9%) after therapy (*P* = 0.014).

#### Relapse

**Fig. 4:**
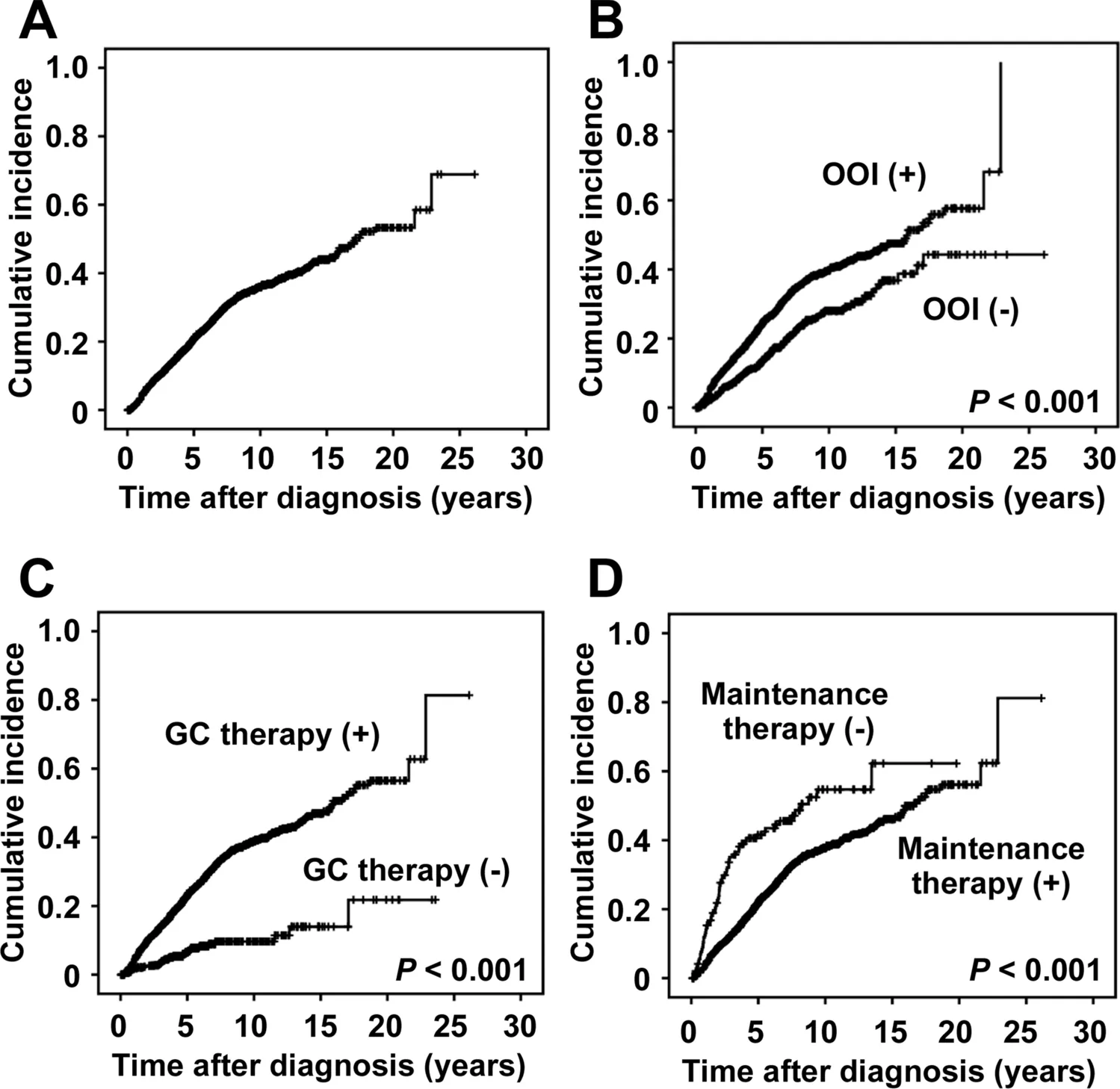
Kaplan–Meier curves for cumulative incidence of relapse after AIP diagnosis. Cumulative incidence of relapse is shown for *A* all patients with AIP, *B* patients stratified by the presence (+) or absence (−) of other organ involvement (OOI) defined in the JPS2018 criteria at diagnosis, *C* patients stratified by the use of initial glucocorticoid (GC) therapy, and *D* patients stratified by the use of maintenance therapy. Tick marks indicate censored cases

Relapse occurred in 787 patients (27.8%) during a median (IQR) follow-up period of 1907 (1273–3014) days after diagnosis. The cumulative incidence of relapse was 12.5%, 20.7%, 36.0%, 44.0%, 53.3%, and 68.9% at 3, 5, 10, 15, 20, and 25 years, respectively (Fig. 4A). Relapse involved the pancreas alone in 58.9%, extrapancreatic organs alone in 30.4%, and both in 10.7% (Supplementary Table 7). Treatment status at relapse was available in 778 patients: no initial GC therapy (*n* = 35), after GC discontinuation (*n* = 333), during GC tapering (*n* = 98), during maintenance GC therapy (*n* = 308), and during other therapies (*n* = 4). Among patients receiving GC therapy at relapse, the median PSL dose was 5 mg/day (IQR 3–5). The cumulative risk of relapse was higher in patients with OOI and in those who received initial GC therapy, and lower in those who received maintenance therapy (all *P* < 0.001) (Fig. 4B–D). Detailed cumulative incidence rates at each time point are summarized in Supplementary Table 8.

#### Malignancies and overall survival

A total of 589 malignancies were identified in 517 patients (18.2%) (Table 2). Multiple malignancies were observed in several patients (four in 2 patients, three in 7, and two in 52). Gastric cancer was the most common malignancy, followed by colorectal, prostate, and bladder cancers. Among 543 malignancies with known timing of diagnosis, 82 (15.1%) were diagnosed within 6 months before or after AIP diagnosis (Supplementary Table 9). Pancreatic cancer was diagnosed in 29 patients. Four cases were diagnosed concurrently with AIP. Pancreatic cancer was diagnosed at 6–12 months, 1–2 years, 2–5 years, 5–10 years, and > 10 years after AIP diagnosis in 2, 3, 7, 9, and 3 patients, respectively. In one patient, pancreatic cancer had been diagnosed 4 years before the diagnosis of AIP. Clinical stage distribution was as follows: IA (*n* = 5), IB (*n* = 3), IIA (*n* = 8), IIB (*n* = 2), III (*n* = 4), and IV (*n* = 6); stage was unknown in 1 patient [14].

**Table 2:** Types of malignancies observed in patients with AIP

Type of malignancy	*n* (%)
Gastric cancer	117 (19.9)
Colorectal cancer	94 (16.0)
Prostate cancer	60 (10.2)
Bladder cancer	58 (9.8)
Lung cancer	57 (9.7)
Breast cancer	33 (5.6)
Malignant lymphoma	31 (5.3)
Pancreatic cancer	29 (4.9)
Kidney cancer	20 (3.4)
Liver cancer	14 (2.4)
Thyroid cancer	9 (1.5)
Ureteral cancer	8 (1.4)
Esophageal cancer	8 (1.4)
Uterine cancer	7 (1.2)
Leukemia	6 (1.0)
Cancer of unknown primary	6 (1.0)
Other malignancies	32 (5.4)
Total	589 (100.0)

Multiple malignancies were observed in several patients (four in 2 patients, three in 7, and two in 52)

**Fig. 5:**
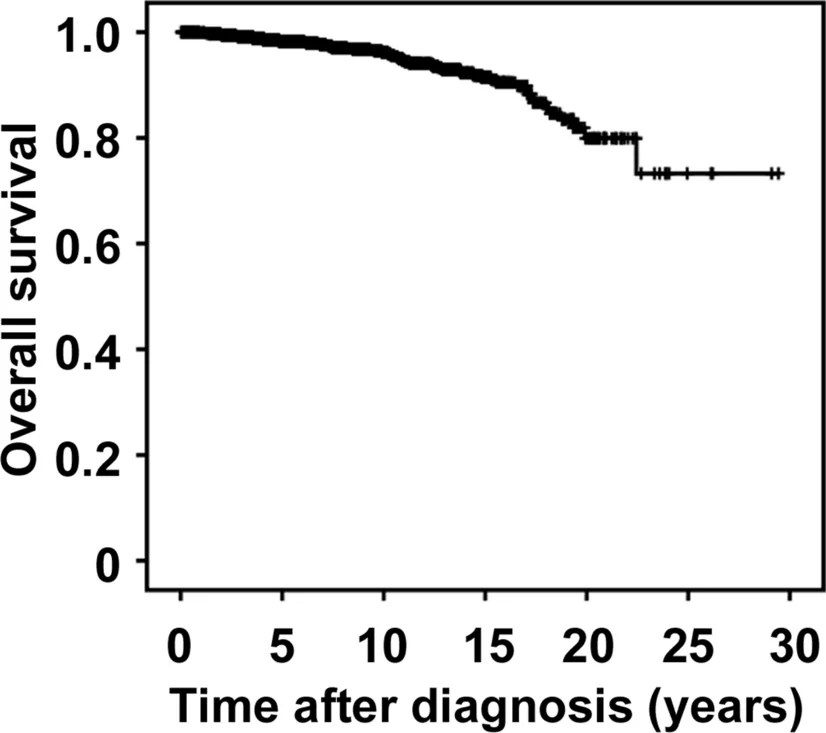
Kaplan–Meier curves for overall survival after AIP diagnosis. Overall survival after AIP diagnosis is shown. Tick marks indicate censored cases

At the time of the survey, 103 patients (3.6%) had died. Causes of death were available for 85 patients; malignancy was the leading cause (40%), followed by infection (28.2%) and cardiovascular disease (11.8%) (Supplementary Table 10). Among the 34 deaths due to malignancy, pancreatic and lung cancers were the most common causes (*n* = 7 each), followed by colorectal cancer (*n* = 4). During a median (IQR) follow-up of 2331 (1459–3744) days, overall survival rates were 99.1%, 98.3%, 96.2%, 91.5%, 79.9%, and 73.3% at 3, 5, 10, 15, 20, and 25 years, respectively **(**Fig. 5**)**.

## Discussion

In this nationwide multicenter study, we estimated the incidence and prevalence of AIP and comprehensively characterized its clinical features in the largest cohort reported to date. By leveraging a nationwide registry across diverse institutions, this study provides a comprehensive real-world overview of AIP in Japan.

The estimated number of patients with AIP in 2021 was 16,750, representing a 1.25-fold increase compared with the 2016 survey. Since the first nationwide survey in 2002, the number of affected patients has increased steadily, approaching a tenfold rise over the past 2 decades. This trend is unlikely to represent a true increase in disease incidence. Rather, it probably reflects improved recognition of AIP and IgG4-RD, refinement of diagnostic criteria, broader acceptance of AIP as a pancreatic manifestation of IgG4-RD, and advances in imaging and endoscopic techniques [1–4, 12, 15]. The diagnostic criteria for AIP and IgG4-RD were revised in 2018 and 2020, respectively [7, 16]. Although population-based data from outside Japan remain limited, reported incidence and prevalence appear lower. In Europe, the annual incidence of AIP has been estimated at 0.29 per 100,000 persons [17], whereas in the United States, the incidence and prevalence of IgG4-RD in 2019 were 1.39 per 100,000 person-years and 5.34 per 100,000 persons, respectively [18]. Nonetheless, similar upward trends have been observed elsewhere, suggesting that enhanced detection and disease awareness, rather than true epidemiological expansion, may underlie these observations globally.

Histopathological evaluation was predominantly performed using EUS-TA. Although now the standard approach, small sample volume and tissue fragmentation may limit assessment of key architectural features such as storiform fibrosis and obliterative phlebitis. Consequently, histological diagnosis of AIP remains challenging in routine practice, as reflected by only slight-to-moderate interobserver agreement among pathologists [19]. To address this limitation, guidance for biopsy-based diagnosis has been proposed [20]. In this study, approximately half of the cases could not be diagnosed histologically based on EUS-TA alone, indicating that the procedure often serves primarily to exclude malignancy, consistent with its role in the JPS2018 criteria [7]. Recent advances, including EUS-guided fine-needle biopsy using new-generation end-cutting needles, have improved the diagnostic yield to over 90% according to ICDC criteria [21] and may further enhance diagnostic performance. Notably, OOI patterns differed by sex. Female patients showed a higher frequency of dacryoadenitis/sialadenitis, whereas sclerosing cholangitis and retroperitoneal fibrosis were more common in male patients. These findings suggest sex-related heterogeneity in the clinical phenotype of AIP/IgG4-RD.

Approximately 16% of patients did not receive initial GC therapy, a proportion comparable to that reported in a large pan-European cohort (13%) [22]. The lower relapse risk observed in these patients most likely reflects lower baseline disease activity rather than a protective effect of conservative management. Current guidelines recommend GC therapy for symptomatic disease, including obstructive jaundice, abdominal pain, back pain, and symptomatic extrapancreatic involvement [15, 23]. Our findings support these recommendations while suggesting that selected asymptomatic patients may be managed conservatively. Consistent with this interpretation, Kubota et al. [24] reported spontaneous remission in 55.7% of patients managed without GC therapy, who generally had milder disease.

Maintenance GC therapy for approximately 3 years is recommended in Japanese guidelines and is widely practiced in Japan [15, 25]. International consensus statements recommend maintenance therapy, particularly for patients at high risk of relapse [26]. In a large European cohort, 47.2% received maintenance therapy, including GC in 26.6% and azathioprine in 12.2% [22]. Meta-analyses have shown that maintenance GC therapy for more than 1 year reduces relapse risk [27]. In our cohort, however, relapse continued to accumulate over decades without an apparent plateau, suggesting that maintenance therapy may delay rather than completely prevent recurrence. These findings support individualized treatment duration and risk-adapted strategies rather than a uniform fixed-term approach. Risk prediction tools, such as the PrescrAIP model, may help identify patients most likely to benefit from prolonged therapy [28].

Long-term GC exposure is associated with substantial adverse effects, including osteoporosis, worsening diabetes, hypertension, infection, and psychological disturbances [29]. Given that AIP predominantly affects older individuals, these complications may substantially impair quality of life and increase overall morbidity [30]. Moreover, pancreatic exocrine insufficiency and diabetes mellitus, both common in AIP, may further contribute to frailty, sarcopenia, and cardiovascular risk [31, 32]. Sano et al. reported a high prevalence of sarcopenia among patients with AIP [33]. Together, these observations underscore the urgent need for effective GC-sparing strategies. Recently, the MITIGATE trial demonstrated that the anti-CD19 monoclonal antibody inebilizumab significantly reduced relapse risk in IgG4-RD, including AIP [34]. In addition, azathioprine and the anti-CD20 monoclonal antibody rituximab have shown efficacy as maintenance therapies [35, 36]. These agents may facilitate safer long-term disease control, particularly in elderly patients or those with recurrent relapse despite conventional maintenance GC therapy.

Long-term overall survival was favorable, exceeding 90% at 15 years. Deaths were attributable primarily to malignancy and infection. Infection was the second leading cause of death, emphasizing the importance of vaccination, infection prevention, and careful monitoring during prolonged immunosuppressive therapy. Although the absolute risk appears low, pancreatic cancer and other malignancies remain important clinical concerns [37, 38]. Proposed mechanisms include inflammation-associated carcinogenesis, paraneoplastic phenomena, and surveillance bias [36]. The reported reduction in pancreatic cancer risk among patients receiving maintenance GC therapy may support a role for persistent inflammation in carcinogenesis [39]. Given that most patients with AIP are elderly, careful surveillance for malignancies, including pancreatic cancer, is warranted. Monitoring should also include extrapancreatic organs, and further studies are needed to define optimal surveillance strategies, including imaging modalities, surveillance intervals, and duration of follow-up, as has also been discussed in chronic pancreatitis [40].

This study has several limitations. First, the response rate was moderate, raising the possibility of selection bias. Second, pathology and imaging findings were not centrally reviewed. Third, treatment effects should be interpreted cautiously because of potential confounding by indication inherent to the observational study design. Finally, international comparisons require caution because of differences in healthcare systems, referral pathways, and diagnostic practices. Nevertheless, the nationwide multicenter design, unprecedented sample size, and comprehensive assessment of long-term outcomes represent major strengths.

## Conclusions

This nationwide survey provides updated epidemiological and clinical data on AIP in Japan. The number of affected patients continues to increase, and the substantial cumulative relapse risk highlights the need for effective steroid-sparing second-line therapies beyond GCs. Further studies focusing on disease mechanisms, predictive biomarkers, and long-term outcomes are needed to optimize patient care.

## Supplementary Information

Below is the link to the electronic supplementary material.

Supplementary file1 (DOCX 2899 KB)

Supplementary file2 (DOCX 43 KB)

## Abbreviations

AIP: Autoimmune pancreatitis
EUS-TA: Endoscopic ultrasonography-guided tissue acquisition
GC: Glucocorticoid
ICDC: International Consensus Diagnostic Criteria
Ig: Immunoglobulin
IgG4-RD: IgG4-related disease
IQR: Interquartile range
JPS2018 criteria: Revised clinical diagnostic criteria for AIP 2018 proposed by the Japan Pancreas Society
MPD: Main pancreatic duct
OOI: Other organ involvement
PSL: Prednisolone
SD: Standard deviation
