# Supplementary material for: Nationwide epidemiological survey of autoimmune pancreatitis in Japan in 2021
Source: J Gastroenterol. 2026 May 19;61(10):1502–11. doi: 10.1007/s00535-026-02438-w (PMC13615955; doi:10.1007/s00535-026-02438-w)
Supplement: Supplementary file 1 [file 535_2026_2438_MOESM1_ESM.docx]

**Supplementary Figure 1. Positive rates of serological markers in patients with AIP.** Elevated serum IgG4 levels (≥135 mg/dL) were observed in 2,535/2,792 patients (90.8%), and elevated serum IgG levels (>1,700 mg/dL) in 1,437/2,621 (54.8%). Antinuclear antibodies (ANA) were positive in 442/1,521 patients (29.1%), and rheumatoid factor (RF) in 202/935 (21.6%).

**Supplementary Figure 2. Distribution of initial daily prednisolone (PSL) doses in patients with AIP.**  (A) Distribution of initial daily PSL doses among 2,253 patients with AIP. Pulse indicates pulse therapy. (B) Body weight data were available for 1,892 patients, and the distribution of initial daily PSL doses per kilogram of body weight is shown.
