# Supplementary material for: Nationwide epidemiological survey of autoimmune pancreatitis in Japan in 2021
Source: J Gastroenterol. 2026 May 19;61(10):1502–11. doi: 10.1007/s00535-026-02438-w (PMC13615955; doi:10.1007/s00535-026-02438-w)
Supplement: Supplementary file 2 [file 535_2026_2438_MOESM2_ESM.docx]

| **Supplementary Table 1. Summary of the first-stage survey** | | | | | |
| --- | --- | --- | --- | --- | --- |
| Strata | Total number of departments | Sampling rate (%) | Number of surveyed departments | Number of responding departments | Response rate (%) |
| University hospitals | 250 | 100 | 250 | 187 | 74.8 |
| Emergency units | 299 | 100 | 299 | 51 | 17.1 |
| ≥500 beds | 514 | 100 | 514 | 212 | 41.2 |
| 400–499 beds | 495 | 80 | 396 | 155 | 39.1 |
| 300–399 beds | 855 | 40 | 342 | 98 | 28.7 |
| 200–299 beds | 862 | 20 | 172 | 65 | 37.8 |
| 100–199 beds | 2,867 | 10 | 286 | 77 | 26.9 |
| ≤99 beds | 2,785 | 5 | 139 | 32 | 23.0 |
| Total | 8,927 | – | 2,398 | 877 | 36.6 |

| **Supplementary Table 2. Clinical presentation at AIP diagnosis** | |
| --- | --- |
| Clinical presentation | n (%) ^a^ |
| Symptomatic ^b^ | 1,648 (58.6) |
| Jaundice | 824 (29.3) |
| Abdominal pain | 672 (23.9) |
| Weight loss | 246 (8.8) |
| Symptoms related to extra-pancreatic lesions | 227 (8.1) |
| Back pain | 196 (7.0) |
| Acute pancreatitis | 60 (2.1) |
| Asymptomatic ^c^ | 1,162 (41.4) |
| Follow-up/work-up of other diseases | 804 (28.6) |
| Medical check-up | 304 (10.8) |
| Clinical presentation was unknown in 23 cases.  ^a^ Among 2,810 cases.  ^b^ Includes overlapping cases.  ^C^ Diagnostic opportunities were unknown in 54 cases. | |

| **Supplementary Table 3. Imaging findings of the pancreas at AIP diagnosis** | |
| --- | --- |
| Pancreatic enlargement, yes, n (%) | 2,819 (99.5) |
| Type of pancreatic enlargement pattern, n (%) ^a^ |  |
| Diffuse (>2/3) | 1,573 (55.8) |
| Segmental (1/3< to <2/3) | 541 (19.2) |
| Focal (<1/3) | 701 (24.9) |
| Atypical | 4 (0.1) |
| MPD narrowing, yes, n (%) ^b^ | 2,424 (88.1) |
| Type of MPD narrowing pattern, n (%) ^c^ |  |
| Diffuse (>2/3) | 1,023 (42.2) |
| Segmental (1/3< to <2/3) | 691 (28.5) |
| Focal (<1/3) | 687 (28.3) |
| Atypical | 23 (0.9) |
| Imaging modality used to assess MPD narrowing, n (%) ^c^ | |
| ERCP only | 265 (10.9) |
| MRCP only | 906 (37.4) |
| Both ERCP and MRCP | 1,203 (49.6) |
| Unknown | 50 (2.1) |
| ^a^ Among 2,819 cases with pancreatic enlargement.  ^b^ Among 2,751 cases assessed for MPD narrowing.  ^c^ Among 2,424 cases with MPD narrowing.  ERCP, endoscopic retrograde cholangiopancreatography; MRCP, magnetic resonance cholangiopancreatography; MPD, main pancreatic duct. | |

| **Supplementary Table 4. Histopathological examinations and findings** | |
| --- | --- |
| Histopathological examination performed, yes, n (%) | 2,340 (84.1) ^a^ |
| Procedure ^b^ |  |
| EUS-TA | 1,973 (85.2) |
| ERCP-guided sampling | 124 (5.4) |
| Percutaneous biopsy | 93 (4.0) |
| Surgical resection | 53 (2.3) |
| Papillary biopsy | 36 (1.6) |
| Other procedures | 38 (1.6) |
| Number of positive histological features ^c^ |  |
| 4 | 152 (6.6) |
| 3 | 381 (16.6) |
| 2 | 583 (25.4) |
| 1 | 395 (17.2) |
| 0 | 782 (34.1) |
| Observed histological features ^c^ | |
| Periductal lymphoplasmacytic infiltration | 1,402 (61.1) |
| Abundant IgG4-positive plasma cells | 1,094 (47.7) |
| Storiform fibrosis | 386 (16.8) |
| Obliterative phlebitis | 430 (18.8) |
| ^a^ Among 2,782 cases.  ^b^ Among 2,317 cases.  ^c^ Among 2,293 cases.  ERCP, endoscopic retrograde cholangiopancreatography;  EUS-TA, endoscopic ultrasonography-guided tissue acquisition. | |

| **Supplementary Table 5. Distribution of OOI by sex** | | | |
| --- | --- | --- | --- |
| Type of OOI | Male, n (%) ^a^ | Female, n (%) ^b^ | *P* value |
| IgG4-related sclerosing cholangitis | 1,095 (50.6) | 268 (41.0) | <0.001 |
| Proximal | 260 (12.0) ^c^ | 46 (7.0) ^d^ | <0.001 |
| Distal | 984 (45.4) ^c^ | 250 (38.3) ^d^ | 0.001 |
| Sialadenitis/dacryoadenitis | 373 (17.2) | 193 (29.6) | <0.001 |
| Sialadenitis | 327 (15.1) ^e^ | 162 (24.8) ^f^ | <0.001 |
| Dacryoadenitis | 126 (5.8) ^e^ | 79 (12.1) ^f^ | <0.001 |
| Retroperitoneal fibrosis | 284 (13.1) | 58 (8.9) | 0.004 |
| IgG4-related kidney disease | 181 (8.4) | 54 (8.3) | 0.94 |
| IgG4-related lung disease | 133 (6.1) | 29 (4.4) | 0.10 |
| Aortitis/periaortitis | 121 (5.6) | 12 (1.8) | <0.001 |
| IgG4-related cholecystitis | 47 (2.2) | 8 (1.2) | 0.13 |
| Pseudotumor | 42 (1.9) | 22 (3.4) | 0.032 |
| IgG4-related liver disease | 21 (1.0) | 4 (0.6) | 0.39 |
| Total cases with any OOI listed in this table | 1,519 (70.1) | 449 (68.8) | 0.53 |
| Total cases with any OOI defined in the JPS2018 criteria | 1,470 (67.9) | 441 (67.5) | 0.87 |
| Number of OOI, mean (SD) ^h^ | 0.89 (0.78) | 0.88 (0.74) | 0.66 |
| ^a^ Among 2,166 cases. ^b^ Among 653 cases. ^c^ Overlap observed in 149 cases. ^d^ Overlap observed in 28 cases.  ^e^ Overlap observed in 80 cases. ^f^ Overlap observed in 48 cases. OOI, other organ involvement; SD, standard deviation. | | | |

| **Supplementary Table 6. Types of immunomodulators** | |
| --- | --- |
| Types of immunomodulator | n (%) |
| Azathioprine | 85 (88.5) |
| Inebilizumab | 3 (3.1) |
| Tacrolimus | 3 (3.1) |
| Rituximab | 2 (2.1) |
| Adalimumab | 1 (1.0) |
| Etanercept | 1 (1.0) |
| Mycophenolate mofetil | 1 (1.0) |
| Total | 96 (100.0) |

| **Supplementary Table 7. Sites of relapse** | |
| --- | --- |
| Sites of relapse ^a^ | n (%) |
| Pancreas | 441 (58.9) |
| Extrapancreatic sites | 228 (30.4) |
| Both pancreatic and extrapancreatic sites | 80 (10.7) |
| Site of extrapancreatic relapse ^b^ |  |
| Bile duct | 220 (71.4) |
| Retroperitoneum | 60 (19.5) |
| Lacrimal/salivary glands | 58 (18.8) |
| Kidney | 56 (18.2) |
| Lung | 22 (7.1) |
| Other sites | 56 (18.2) |
| ^a^ Site of relapse was reported in 749 cases.  ^b^ Among 308 cases, with overlapping sites allowed. | |

| **Supplementary Table 8. Cumulative incidence of relapse in patients with AIP** | | | | | | | | |
| --- | --- | --- | --- | --- | --- | --- | --- | --- |
| Time after AIP diagnosis | Cumulative incidence of relapse, % | | | | | | | |
|  | OOI at AIP diagnosis * | |  | Initial GC therapy | |  | Maintenance therapy | |
|  | Presence | Absence |  | Presence | Absence |  | Presence | Absence |
| 3 years | 15.0 | 7.4 |  | 14.1 | 3.4 |  | 12.8 | 34.5 |
| 5 years | 24.2 | 13.4 |  | 23.1 | 6.5 |  | 22.0 | 40.9 |
| 10 years | 39.8 | 28.1 |  | 39.7 | 9.7 |  | 38.8 | 53.8 |
| 15 years | 47.5 | 36.8 |  | 47.8 | 15.2 |  | 47.0 | 61.5 |
| 20 years | 57.6 | 44.3 |  | 57.2 | 22.9 |  | 56.8 | NR |
| 25 years | 100.0 | 44.3 |  | 81.7 | NR |  | 81.5 | NR |
| Cumulative incidence was estimated using the Kaplan–Meier method.  * OOI defined in the JPS2018 criteria. GC, glucocorticoid; NR, not reached; OOI, other organ involvement. | | | | | | | | |

| **Supplementary Table 9. Temporal relationship between malignancy diagnosis and AIP diagnosis** | |
| --- | --- |
| Timing of malignancy diagnosis | n (%) |
| Before AIP diagnosis  > 10 years  > 5 to *≤* 10 years  > 2 to ≤ 5 years  > 6 months to ≤ 2 years | 230 (42.4)  63 (11.6)  54 (9.9)  69 (12.7)  44 (8.1) |
| Within 6 months before or after AIP diagnosis | 82 (15.1) |
| After AIP diagnosis  > 6 months to ≤ 2 years  > 2 to *≤* 5 years  > 5 to ≤ 10 years  > 10 years | 231 (42.5)  64 (11.8)  59 (10.9)  69 (12.7)  39 (7.2) |
| Total | 543 (100) |
| Timing of malignancy diagnosis was unknown for 46 malignancies. | |

| **Supplementary Table 10. Causes of death** | |
| --- | --- |
| Cause | n (%) |
| Malignant diseases | 34 (40.0) |
| Pancreatic cancer | 7 (8.2) |
| Lung cancer | 7 (8.2) |
| Colorectal cancer | 4 (4.7) |
| Gastric cancer | 3 (3.5) |
| Prostate cancer | 3 (3.5) |
| Esophageal cancer | 2 (2.4) |
| Malignant lymphoma | 2 (2.4) |
| Other malignancies | 6 (7.1) |
| Infection | 24 (28.2) |
| Pneumonia | 11 (12.9) |
| Coronavirus disease 2019 | 7 (8.2) |
| Sepsis | 2 (2.4) |
| Cholangitis | 2 (2.4) |
| Infection of pancreatic cyst | 2 (2.4) |
| Cardiovascular diseases | 10 (11.8) |
| Acute myocardial infarction | 4 (4.7) |
| Rupture of abdominal aortic aneurysm | 2 (2.4) |
| Other cardiovascular diseases | 4 (4.7) |
| Cerebrovascular diseases | 5 (5.9) |
| Cerebral infarction | 3 (3.5) |
| Other cerebrovascular diseases | 2 (2.4) |
| Liver failure | 3 (3.5) |
| Old age | 3 (3.5) |
| Other causes | 6 (7.1) |
| Total | 85 (100.0) |
| Cause of death was unknown for 18 cases. | |
